# Multi-Trait Screening Identifies Cold-Adapted Soybean Rhizobia from Northeastern China as Candidate Inoculant Strains

**DOI:** 10.3390/plants15152251

**Published:** 2026-07-23

**Authors:** Jinsheng Wang, Li Ma, Guofeng Pu, Jiajun Wang, Ruiping Zhang, Junjiang Wu

**Affiliations:** Key Laboratory of Soybean Cultivation of Ministry of Agriculture, Soybean Research Institute, Heilongjiang Academy of Agricultural Sciences, Harbin 150086, China; jinshengwang1981@163.com (J.W.); mali10291314@163.com (L.M.); puguofeng@sohu.com (G.P.); junjiawang@163.com (J.W.); ruiping_zhang121@126.com (R.Z.)

**Keywords:** *Bradyrhizobium*, biological nitrogen fixation, plant growth-promoting rhizobacteria (PGPR), abiotic stress tolerance, phenotypic diversity, sustainable agriculture, soybean inoculants

## Abstract

Developing effective rhizobial inoculants for high-latitude soybean production requires strains that are both well-adapted to local soil conditions and capable of sustaining symbiotic nitrogen fixation under abiotic stress. In this study, 66 indigenous rhizobial strains isolated from four ecological regions of Heilongjiang Province, China, i.e., the Songnen Plain, Sanjiang Plain, Northwest Arid Sandy Region, and Daxing’anling–Xiaoxing’anling mountainous area, were characterized for phenotypic diversity, abiotic stress tolerance, symbiotic nitrogen fixation capacity, and plant growth-promoting (PGPR) traits. Carbon and nitrogen source utilization, physiological and biochemical properties, and tolerance to salinity, pH extremes, temperature, and drought were assessed and analyzed by hierarchical cluster analysis. The strains showed habitat-associated phenotypic differentiation, with stress-tolerance traits accounting for the greatest proportion of variation. Isolates from the Northwest Arid Sandy Region displayed the broadest stress tolerance, while Sanjiang Plain isolates showed more conservative phenotypic profiles. Eight representative strains were subsequently evaluated for symbiotic performance and PGPR activity. Nodule dry weight, nitrogenase activity, and total plant nitrogen content were strongly intercorrelated and appeared more reliable indicators of nitrogen-fixation efficiency than nodule number alone. Songnen Plain strains SN1 and SN8 showed the highest symbiotic performance, while Northwest Arid Sandy Region strain FS1 exhibited the greatest ACC deaminase activity. A two-dimensional ranking framework integrating symbiotic and non-symbiotic traits ordered the strains as SN1 > SN8 > SJ1 > SJ11 > DX5 > DX1 > FS7 > FS1, identifying SN1 and SN8 as the most promising candidates requiring field validation for inoculant applications. These findings suggest that multi-trait evaluation frameworks may offer a practical and reproducible approach to screening indigenous rhizobia for region-specific biofertilizer applications in cold-region soybean agriculture.

## 1. Introduction

Soybean (*Glycine max* L.) is among the most economically important crops in China, supplying a major share of dietary protein and vegetable oil for food, feed, and industrial uses [1,2]. Nitrogen availability is generally considered the most limiting factor for soybean productivity, influencing vegetative growth, reproductive development, and yield [3]. Production systems remain heavily reliant on synthetic nitrogen inputs, and sustained over-application has been associated with soil compaction, declining fertility, non-point-source pollution, and elevated greenhouse gas emissions [4,5], raising concerns about the long-term sustainability of current fertilization practices.

Biological nitrogen fixation through the soybean–rhizobia symbiosis represents one of the more efficient nitrogen-acquisition pathways available in agricultural systems [6,7,8]. This symbiosis converts atmospheric dinitrogen into plant-usable ammonium without external nitrogen inputs and may help reduce fertilizer dependence while supporting rhizosphere nutrient cycling [9,10]. Beyond symbiotic fixation, many rhizobia also act as plant growth-promoting rhizobacteria (PGPR), contributing to phosphate solubilization, auxin biosynthesis, and ACC deaminase activity, traits that can jointly improve nutrient acquisition, modulate hormonal balance, and alleviate abiotic stress in the host plant [11,12].

Heilongjiang Province, as China’s principal high-latitude soybean-producing region [13], spans four agroecological zones—the Songnen Plain, the Sanjiang Plain, the northwest arid sandy region, and the Daxing’anling–Xiaoxing’anling mountainous area—that differ substantially in temperature regime, precipitation, and soil properties. These contrasting conditions, which include prolonged cold exposure, intermittent drought, and variable salinity and nutrient status, may have imposed differential selective pressures on indigenous rhizobial populations over time, potentially contributing to regional differentiation in phenotypic traits, stress tolerance, and symbiotic performance. Strains that have persisted under local conditions for extended periods could, in principle, offer greater environmental resilience and host compatibility than non-native inoculant strains, making them relevant candidates for region-specific inoculant development [14].

Despite progress in characterizing soybean rhizobial diversity in temperate and tropical settings [15,16], comparatively little work has examined cold-adapted, high-latitude rhizobial populations using an integrated multi-trait approach. Much of the existing literature has emphasized either genetic diversity or isolated functional assays, with fewer studies combining systematic phenotypic profiling, verified symbiotic activity, and quantitative growth-promotion data within a single evaluation framework. A further limitation is the continued reliance on nodule number as a primary screening criterion, even though this metric appears to correlate poorly with actual nitrogen-fixation efficiency. In the absence of a standardized, multi-trait screening approach suited to high-latitude conditions, identifying elite Heilongjiang rhizobial strains for practical application remains difficult.

To address this gap, the present study examined 66 indigenous soybean rhizobial strains isolated from the four major ecological regions of Heilongjiang Province. The specific objectives were to (i) characterize phenotypic diversity across carbon and nitrogen source utilization, stress tolerance, and physiological/biochemical traits; (ii) assess patterns of phenotypic differentiation by hierarchical cluster analysis; (iii) evaluate symbiotic nodulation and nitrogen-fixation capacity through pot experiments; (iv) quantify non-symbiotic plant growth-promoting traits, including phosphate solubilization, IAA synthesis, ACC deaminase activity, and siderophore production; and (v) develop a two-dimensional evaluation framework integrating symbiotic and non-symbiotic data to support the identification of candidate strains suited to cold-region agricultural environments.

## 2. Results

### 2.1. Carbon and Nitrogen Source Utilization Rates

The 66 strains showed considerable variation in carbon and nitrogen source utilization, suggesting substantial metabolic diversity within the collection (Table 1). Most strains utilized common monosaccharides, disaccharides, and organic acid salts, although utilization of inositol and lactose was more variable and provided some discrimination among strains. Similarly, most strains were able to use arginine and phenylalanine as sole nitrogen sources, whereas utilization of L-histidine was lower and more variable across regions. Songnen Plain strains showed broad, relatively balanced utilization of most carbon and nitrogen substrates, consistent with the nutrient-rich character of black soil environments. Sanjiang Plain strains had comparatively lower utilization rates for several carbon sources, particularly lactose (72.73%), potentially reflecting more conservative metabolic strategies associated with wetland habitats. Northwest arid-region strains showed the highest utilization rates across nearly all substrates tested, suggesting broader metabolic flexibility as an adaptation to nutrient-limited or stressful conditions. Mountain area strains showed intermediate but more heterogeneous utilization patterns, consistent with the diversity of oligotrophic mountain habitats.

### 2.2. Stress Tolerance

All strains grew under 0.5–1.5% NaCl; tolerance declined progressively at higher concentrations, with marked inter-strain differentiation at 2.0% and 2.5% NaCl (Table 2). No strain grew at 4 °C; optimal growth occurred at 28 °C. High temperature (45 °C) was inhibitory to most strains. The optimal pH range was 5.0–9.0, with strong growth across all strains at pH 6.0–8.0; pH 4.0 and 10.0 were strongly inhibitory. PEG-6000 at 20% clearly differentiated strains by drought tolerance, and 30% was lethal for nearly all strains. Northwest arid region strains showed the highest tolerance proportions under high salinity (42.9% at 2.5% NaCl), extreme pH (57.1% at pH 4.0; 71.4% at pH 10.0), high temperature (21.4% at 45 °C), and severe drought (35.7% at 25% PEG-6000), consistent with prolonged multi-stress adaptation. Sanjiang Plain strains were comparatively more sensitive to all extreme conditions, consistent with the mild, stable wetland environment of their origin. Mountain area strains were distinguished by comparatively higher proportions of low-temperature-tolerant strains (29.6% at 10 °C vs. 8.7–14.3% in other regions). Songnen Plain strains exhibited intermediate, broadly balanced stress-tolerance profiles.

### 2.3. Physiological and Biochemical Characteristics

All 66 strains were catalase-positive, indicating a universal capacity to decompose hydrogen peroxide (Table 3). Most strains were positive for the BTB alkaline reaction and were citrate-utilization-positive, reflecting a widespread capacity to utilize organic acid substrates. Starch hydrolysis and gelatin liquefaction rates were intermediate and showed clear regional differentiation. Indole production, hydrogen sulfide production, and the VP reaction were positive in only a minority of strains across all regions. Northwest arid-region strains had the highest positive rates of extracellular enzyme activities and specialized metabolic reactions, consistent with greater metabolic versatility under nutrient-limited conditions. Sanjiang Plain strains showed the most conservative profiles across all tests. Mountain area strains exhibited the greatest variation among individuals, consistent with heterogeneous habitat conditions.

### 2.4. Cluster Analysis, Phenotypic Differentiation, and Association with Ecological Origin

**Figure 1:**
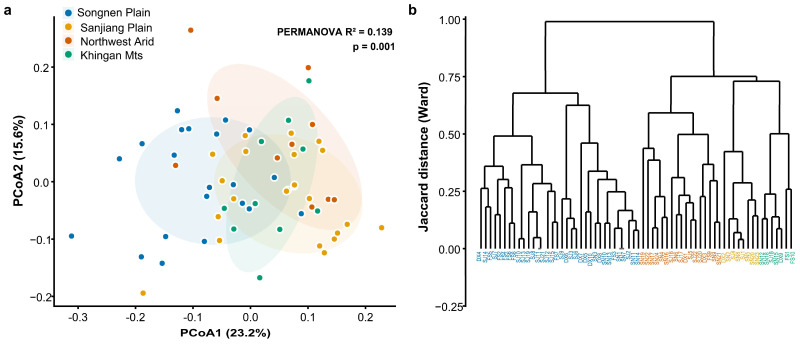
Phenotypic dissimilarity among 66 soybean rhizobial strains from four ecological regions, assessed by principal coordinate analysis and hierarchical clustering. (**a**) Principal coordinate analysis (PCoA) based on Jaccard distance, with strains colored by ecological region of origin (Songnen Plain, Sanjiang Plain, Northwest Arid Sandy, Mountain Area). Shaded ellipses denote 95% confidence regions for each group; inset values show PERMANOVA R^2^ and *p*-value. (**b**) Hierarchical clustering dendrogram of the same Jaccard distance matrix using the UPGMA (average-linkage) method; strain labels are colored by ecological region as in (**a**).

Principal coordinate analysis (PCoA) based on Jaccard distance indicated some separation of strains according to ecological region of origin, with PCoA1 and PCoA2 accounting for 23.2% and 15.6% of total variance, respectively (Figure 1a). PERMANOVA indicated that ecological region accounted for a modest, statistically significant proportion of overall phenotypic dissimilarity (R^2^ = 0.139, *p* = 0.001), suggesting region is associated with part of the observed structure, although most variance remained unexplained by region alone. The 95% confidence ellipses for the four regions overlapped substantially, particularly among Sanjiang Plain, Northwest Arid Sandy, and Mountain Area strains, while Songnen Plain strains tended to occupy somewhat more negative values along PCoA1.

Hierarchical clustering of the same dissimilarity matrix using the UPGMA (average-linkage) method (Figure 1b) likewise did not separate strains into clades that cleanly corresponded to ecological region; strains from all four regions appeared interspersed across the dendrogram rather than forming distinct, region-specific branches. This pattern is consistent with the modest PERMANOVA R^2^ observed in the PCoA and suggests that, while ecological origin contributes to some of the variation among strains, it is not the dominant factor structuring overall phenotypic similarity in this collection.

**Figure 2:**
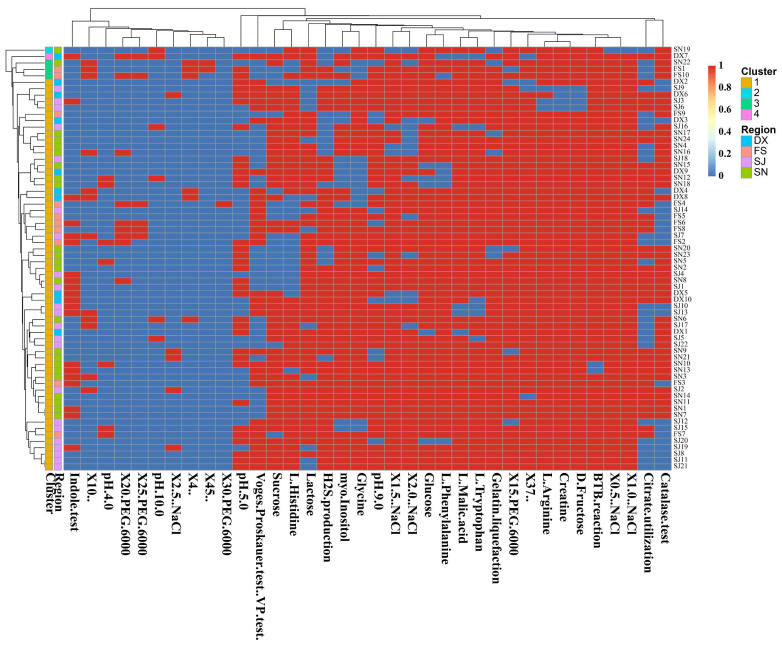
Heatmap of binary phenotypic trait profiles for 66 soybean rhizobial strains, with two-way hierarchical clustering of strains and traits. Rows correspond to strains, ordered according to the dendrogram at left; columns correspond to phenotypic indicators (carbon and nitrogen source utilization, stress tolerance, and physiological/biochemical tests), ordered according to the dendrogram at top. Cell color indicates a positive (1, red) or negative (0, blue) result for each strain–trait combination, shown on a continuous color scale. Left-hand sidebars indicate cluster assignment (clusters 1–4) and ecological region of origin (DX, Mountain Area; FS, Northwest Arid Sandy; SJ, Sanjiang Plain; SN, Songnen Plain) for each strain.

Heatmap visualization of the full binary phenotypic trait matrix, annotated by cluster assignment and ecological region (Figure 2), showed that most strains fell within a single large cluster, with smaller numbers forming separate, more distinct clusters; as with the PCoA and dendrogram, ecological region did not segregate cleanly along these cluster boundaries. Across traits, carbon and nitrogen source utilization together with several core physiological tests (e.g., catalase activity, citrate utilization, tolerance to low NaCl concentrations) showed consistently high positive rates across nearly all strains, suggesting this represents a relatively conserved portion of the phenotypic profile. In contrast, traits reflecting tolerance to more extreme stress conditions (e.g., high-concentration NaCl, extreme pH, high PEG concentrations) and several specialized biochemical reactions (e.g., indole and hydrogen sulfide production) showed more variable, strain-specific patterns and appear to account for most of the observed inter-strain discrimination. Together, these results suggest that phenotypic differentiation among strains is driven primarily by variation in stress-tolerance and specialized biochemical traits rather than by core metabolic capacity, and that this differentiation is only partially explained by the ecological region of origin.

### 2.5. Selection of Representative Strains

Eight representative strains were selected for subsequent functional characterization, based on the cluster assignments derived from the phenotypic clustering analysis (Figure 1 and Figure 2). Two strains were selected per cluster, yielding eight strains total, with selection further constrained so that each of the four ecological regions (Songnen Plain, Sanjiang Plain, Northwest Arid Sandy, Mountain Area) contributed exactly two strains: SN1 and SN8 (Songnen Plain), SJ1 and SJ11 (Sanjiang Plain), FS1 and FS7 (Northwest Arid Sandy), and DX1 and DX5 (Mountain Area). Within each cluster, strains were chosen to occupy distinct positions in the dendrogram rather than adjacent terminal branches, with the aim of capturing a reasonable range of within-cluster phenotypic variation rather than near-identical profiles. This selection strategy was intended to balance representation across the cluster structure and to ensure coverage of the four sampled ecological regions, providing a basis for comparisons of symbiotic and plant growth-promoting traits.

### 2.6. Symbiotic Nitrogen Fixation Performance

#### 2.6.1. Nodulation and Plant Growth

**Figure 3:**
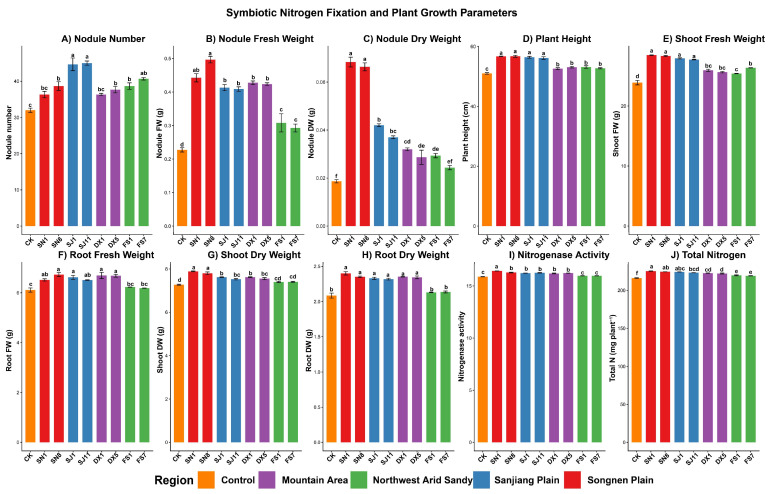
Symbiotic nitrogen fixation and plant growth parameters for eight representative rhizobial strains and uninoculated control (CK). (**A**) Nodule number; (**B**) Nodule fresh weight; (**C**) Nodule dry weight; (**D**) Plant height; (**E**) Shoot fresh weight; (**F**) Shoot dry weight; (**G**) Root fresh weight; (**H**) Root dry weight; (**I**) Nitrogenase activity; (**J**) Total plant nitrogen content. Error bars represent the standard error of the mean (SEM) across three biological replicates. Significance letters are placed above error bars. Different letters indicate significant differences at *p* < 0.05 (Duncan’s test).

All eight strains formed effective symbiotic associations with soybean (Figure 3). Nodule number differed significantly among strains: SJ11 had the highest nodule count, followed by SJ1; these two did not differ significantly from each other but exceeded most other strains. FS7, FS1, and SN8 were intermediate. SN1, DX1, and DX5 produced fewer nodules but remained significantly above the control level. Nodule fresh weight was highest in SN8, significantly exceeding that of all other strains. Nodule dry weight was highest for SN1 and SN8 collectively, followed by SJ1 and SJ11; FS1, FS7, DX1, and DX5 showed a descending gradient, all significantly above the control. Plant height was significantly increased by all strains relative to the control; SN1, SN8, SJ1, and SJ11 showed the greatest height promotion, while FS1, FS7, DX1, and DX5 were not significantly different from each other but were significantly lower than the first group. Shoot and root biomass indicators showed similar patterns, with SN1 and SN8 generally producing the highest shoot biomass, and SN1, SN8, DX1, and DX5 showing the highest root dry weights.

#### 2.6.2. Nitrogenase Activity and Total Plant Nitrogen

SN1 had significantly higher nitrogenase activity than all other strains. SN8, SJ1, SJ11, and DX5 did not differ significantly from each other but were significantly higher than DX1, FS1, and FS7. FS1 and FS7 had the lowest nitrogenase activities among inoculated treatments, though both remained significantly above the control (Figure 3). Total plant nitrogen content followed the same overall pattern: SN1, SN8, and SJ1 formed the highest-ranking group; SJ11, DX1, and DX5 formed a middle tier; FS1 and FS7 were lowest among inoculated treatments but significantly exceeded the control.

#### 2.6.3. Correlations Among Symbiotic Traits

**Figure 4:**
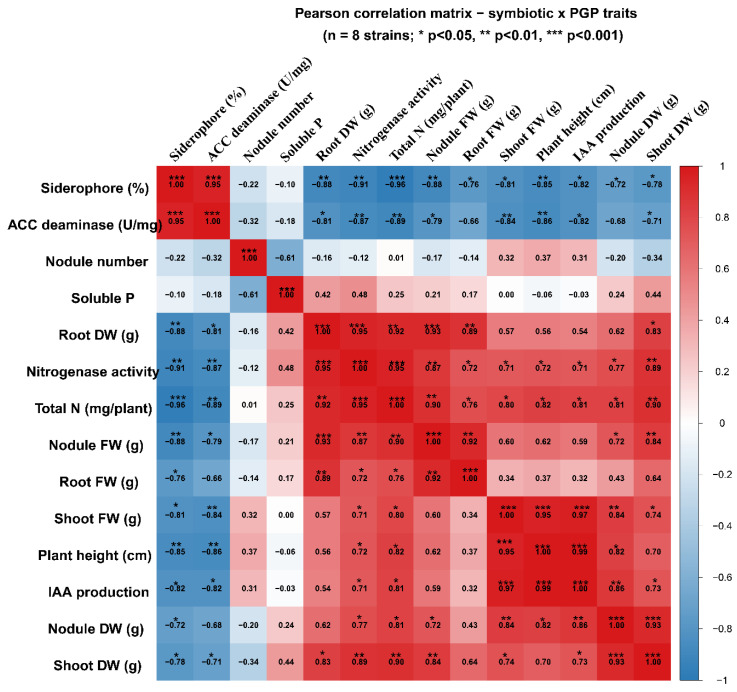
Pearson correlation matrix among nodule traits, plant growth parameters, and nitrogen indicators for soybean inoculated with eight rhizobial strains. Upper triangle: correlation coefficients (r). Lower triangle: significance levels. * *p* < 0.05; ** *p* < 0.01; *** *p* < 0.001.

Pearson correlation analysis of the symbiotic trait dataset revealed strong positive associations among nodule biomass, plant growth, and nitrogen fixation indicators, while nodule number was largely uncorrelated with most functional traits (Figure 4). Nodule fresh weight and dry weight were strongly correlated with each other. Nodule number, however, correlated poorly with nodule fresh weight and dry weight, suggesting that nodule count does not reliably reflect nodule quality or nitrogen fixation potential. Nitrogenase activity was strongly associated with nodule fresh weight, nodule dry weight, shoot dry weight, root dry weight, and total plant nitrogen, consistent with nodule biomass serving as a key determinant of nitrogen fixation efficiency. Total plant nitrogen and root dry weight were also strongly correlated. In contrast, nodule number correlated weakly with nitrogenase activity and total nitrogen. These results suggest that nodule dry weight, nitrogenase activity, and total plant nitrogen content may be more appropriate primary screening criteria for Heilongjiang soybean rhizobia than nodule number.

#### 2.6.4. Association of Symbiotic Traits with Phenotypic Group and Ecological Origin

**Figure 5:**
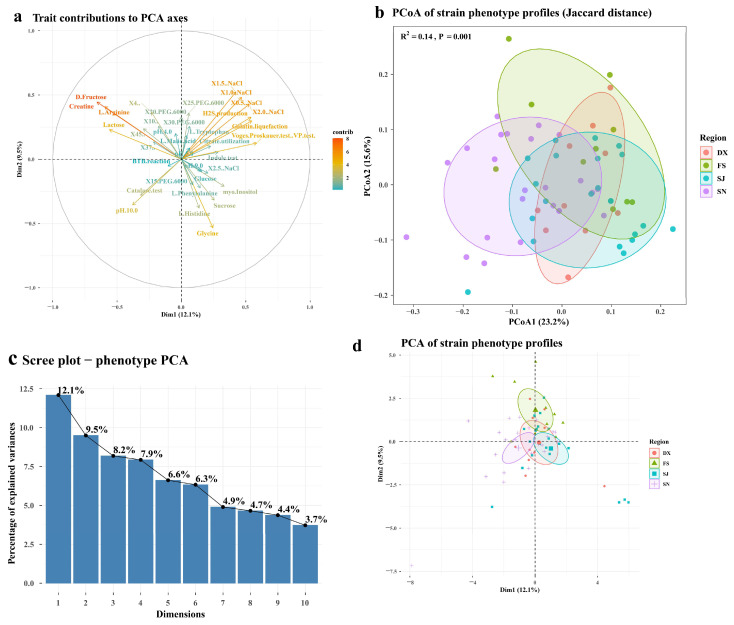
Ordination analyses of phenotypic trait variation among soybean rhizobial strains. (**a**) Loading plot showing the contribution of individual phenotypic traits to the first two principal component (PCA) axes; arrow length and color indicate the relative magnitude of each trait’s contribution. (**b**) Principal coordinate analysis (PCoA) of strain phenotype profiles based on Jaccard distance, with strains colored by ecological region of origin and shaded ellipses denoting group dispersion; inset values show the PERMANOVA R^2^ and associated *p*-value. (**c**) Scree plot showing the percentage of variance explained by each of the first ten principal component axes derived from the phenotypic trait matrix. (**d**) PCA ordination of strain phenotype profiles, colored by ecological region of origin, with shaded ellipses denoting group dispersion.

PCA of the eight representative strains showed that the distribution of strains in the ordination space corresponded simultaneously to phenotypic group membership and ecological origin (Figure 5a–d), suggesting that both genetic background and habitat history may contribute to the expression of symbiotic functional traits. Full PCA loading values and derived weights for all seven indicators are provided in Supplementary Tables S1–S3. Strains sharing both phenotypic group and ecological origin showed the most similar symbiotic profiles. SJ1 and SJ11, sharing closely related phenotypic backgrounds and wetland-habitat origins, showed similar nodulation phenotypes; SN1 and SN8, as members of the same major phenotypic group, demonstrated stable, high symbiotic performance. Strains from the northwest arid and mountain regions showed greater inter-strain functional dispersion, consistent with their more heterogeneous environments. Symbiotic performance broadly followed the gradient: Songnen Plain > Sanjiang Plain > Mountain Area > Northwest Arid Region, consistent with a possible resource allocation trade-off between stress tolerance and symbiotic efficiency, though longitudinal evidence would be required to confirm this interpretation.

### 2.7. Non-Symbiotic Plant Growth-Promoting Traits

#### 2.7.1. Single-Indicator Functional Differentiation

**Figure 6:**
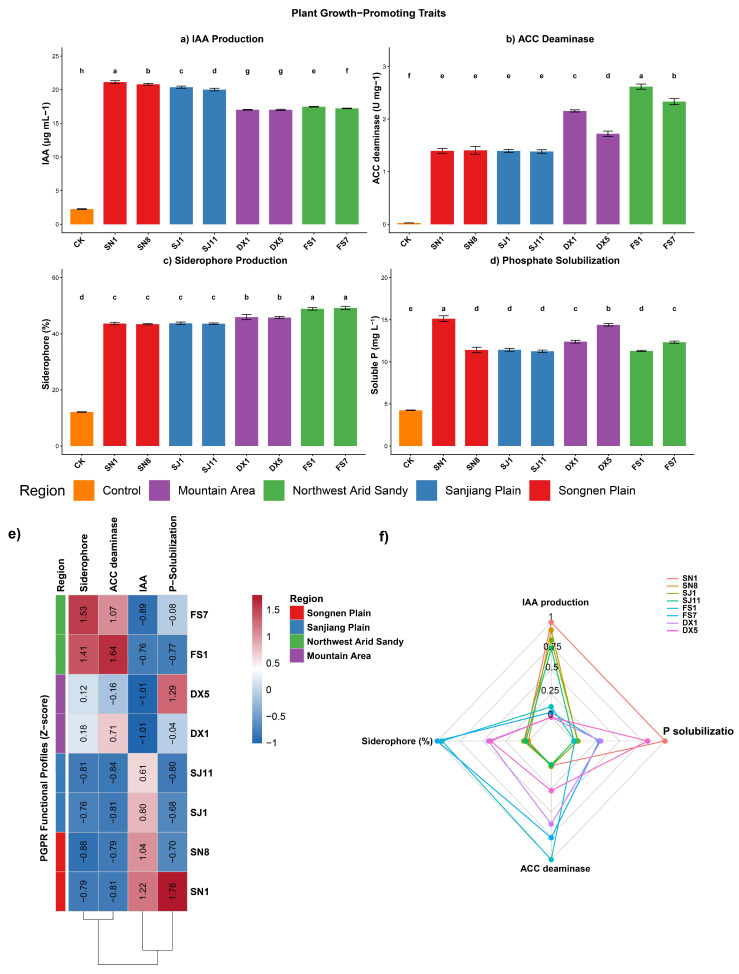
Non-symbiotic plant growth-promoting (PGPR) functional traits of eight representative soybean rhizobial strains. (**a**): bar plots (mean ± SD) for ACC deaminase activity (U mg^−1^ protein), (**b**): IAA production (µg mL^−1^), (**c**): relative siderophore production rate (%), (**d**): inorganic phosphate solubilization capacity (mg soluble P L^−1^). (**e**): z-score heatmap of the four PGPR indicators for the eight representative strains. Rows represent strains arranged from top (SN1) to bottom (DX5); columns represent the four PGPR traits. Color scale ranges from −2 (blue, below group mean) to +2 (red, above group mean); white indicates values close to the group mean. Numeric values within cells show the z-score for each strain–trait combination. The colored sidebar indicates the ecological region of origin. (**f**): radar (spider) chart of min–max normalized PGPR indicator values (0–1 scale) for all eight representative strains and the uninoculated control. Each polygon represents one strain; axis labels indicate the four PGPR traits. Lines are colored by strain identity as indicated in the legend. Note: radar plot polygon area is a function of both trait magnitude and axis number; visual area differences should not be interpreted as proportional to biological differences.

All eight strains significantly exceeded the uninoculated control (CK) for all four PGPR indicators (Figure 6). The rank order of strains differed substantially among the four traits, indicating that PGPR functional capacity is not uniformly distributed but reflects trait-specific ecological specialization. IAA production was highest in Songnen Plain strains (Figure 6a). SN1 produced significantly more IAA than all other strains, followed by SN8, SJ1, and SJ11. DX1 and DX5 produced markedly less, forming the lowest group among inoculated strains along with FS7 and FS1. CK had the lowest IAA of all groups. This pattern, Songnen Plain and Sanjiang Plain strains ranking highest, and Northwest Arid Sandy Region and mountain area strains lowest, is the inverse of the stress tolerance gradient observed in the phenotypic characterization. ACC deaminase activity showed the clearest gradient among the four PGPR traits (Figure 6b). FS1 had the highest enzyme activity, followed significantly by FS7, DX1, and DX5. SN1, SN8, SJ1, and SJ11 all fell in the lowest group and did not differ significantly from each other, though all remained significantly above CK. The five-group stratification for ACC deaminase was the most finely resolved of any single PGPR indicator, reflecting a clear ecological gradient from the stress-adapted Northwest Arid Sandy Region and mountain area strains to the symbiosis-oriented Songnen Plain and Sanjiang Plain strains. Siderophore relative production rate followed the opposite regional pattern to IAA (Figure 6c). FS7 and FS1 were not significantly different. DX1 and DX5 formed an intermediate group. SN1, SN8, SJ1, and SJ11 had the lowest siderophore production among inoculated strains, with SN8 and SJ11 at near-zero normalized values, and CK was the lowest of all. The co-occurrence of high siderophore production with high ACC deaminase activity in FS strains, and near absence of both traits in SN and SJ strains, is consistent with a functionally integrated stress-mitigation phenotype in Northwest Arid Sandy Region strains.

Inorganic phosphate solubilization showed a distinct stratification pattern that did not align with either the IAA or the siderophore/ACC deaminase groupings (Figure 6d). SN1 significantly released the most soluble phosphorus of all strains. DX5 ranked second, substantially above all remaining strains. DX1 and FS7 were intermediate and not significantly different from each other. SN8, SJ1, SJ11, and FS1 formed the lowest-inoculated group, with CK the lowest of all. Phosphate solubilization is the only PGPR trait for which a mountain area strain (DX5) ranked among the top two performers alongside a Songnen Plain strain (SN1), and the only trait for which FS1 ranked near the bottom despite being the top ACC deaminase producer.

#### 2.7.2. Integrated PGPR Functional Profiles

Z-score heatmap analysis of the four normalized PGPR indicators (Figure 6e) revealed distinct functional profiles for each strain and confirmed the complementary nature of trait expression across ecological groups. SN1 displayed strongly positive z-scores for IAA production and soluble phosphorus content, and strongly negative z-scores for ACC deaminase activity and siderophore production, defining a profile oriented toward auxin-mediated growth promotion and phosphorus mobilization. SN8 showed a similar profile to SN1, with a high IAA z-score but a near-zero soluble P z-score. SJ1 and SJ11 shared a profile dominated by positive IAA z-scores, with near-zero or negative scores for the other three traits, and clustered phenotypically close to the SN strains in the PGPR functional space. FS1 showed the most extreme functional profile: strongly positive z-scores for both ACC deaminase activity and siderophore production, and strongly negative z-scores for IAA and soluble phosphorus, the functional mirror image of SN1. FS7 showed a profile like FS1, with the highest siderophore z-score in the dataset. DX1 had moderately positive z-scores for ACC deaminase and siderophore, with near-zero IAA and phosphorus values, representing an intermediate stress-mitigation profile. DX5 was the most functionally balanced strain: moderately positive z-scores for soluble phosphorus and ACC deaminase, with intermediate siderophore and near-zero IAA values, and no strongly negative scores on any indicator.

#### 2.7.3. Comprehensive PGPR Evaluation by Radar Plot

Radar plot analysis of the four normalized PGPR indicators (Figure 6f) was consistent with functional polarization between ecological groups and indicated that DX5 showed the most balanced multi-trait PGPR profile among the eight strains evaluated. SN1 produced the largest radar polygon overall, driven by its high scores on the IAA and soluble phosphorus axes; however, its polygon was markedly asymmetric, with near-zero values on the siderophore and ACC deaminase axes. SN8 showed a similar but slightly smaller asymmetric polygon. SJ1 and SJ11 produced intermediate-sized polygons dominated by the IAA axis, with limited contributions from the other three traits. FS1 produced a polygon with a relatively small total area, with elevated contributions from siderophores and ACC deaminase but near-zero IAA and soluble phosphorus scores, consistent with a narrow, stress-oriented functional profile. FS7 showed a siderophore-dominated polygon like FS1 but with a marginally higher ACC deaminase contribution. DX1 contributed moderate values only on the ACC deaminase axis. DX5 produced the most geometrically balanced polygon among all strains, with meaningful contributions on three of four axes (ACC deaminase, siderophore, and soluble phosphorus) and no axis at near-zero, consistent with its identification as the most functionally balanced PGPR strain among those evaluated.

### 2.8. Integrated Functional Profiles from Z-Score Heatmaps

**Figure 7:**
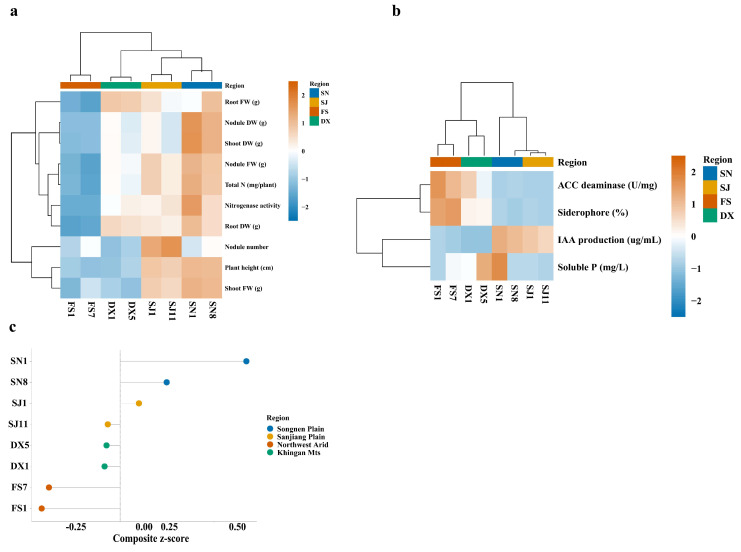
Composite evaluation of symbiotic and growth-promoting performance among eight representative rhizobial strains, integrating nodulation, plant growth, and PGPR traits with an overall ranking score. (**a**) Heatmap of standardized (z-score) values for nodule and plant growth parameters, including nodule number, nodule and shoot/root fresh and dry weight, plant height, nitrogenase activity, and total nitrogen content across the eight strains, with strains and traits hierarchically clustered and ecological region of origin indicated above. (**b**) Heatmap of standardized values for plant growth-promoting traits (ACC deaminase activity, siderophore production, IAA production, and soluble phosphorus release), clustered and annotated as in (**a**). (**c**) Composite z-scores derived from the two-dimensional evaluation framework, ranking the eight strains from highest to lowest and colored by ecological region of origin. Line weights and styles have been revised for improved legibility.

Z-score normalization of symbiotic indicators (Figure 7a) indicated that SN1 and SN8 showed consistently positive z-scores across all symbiotic traits root fresh weight, nodule dry weight, shoot dry weight, nodule fresh weight, total nitrogen, nitrogenase activity, root dry weight, nodule number, plant height, and shoot fresh weight while FS1 and FS7 showed predominantly negative (cool) z-scores across the same indicators, with the largest deficits in nitrogenase activity, total nitrogen, and root dry weight. SJ1, SJ11, DX1, and DX5 occupied intermediate positions, with most z-scores close to zero. The heatmap suggested that nitrogenase activity and total plant nitrogen were the traits most clearly associated with the contrast between high- and low-performing strains. The PGPR z-score heatmap (Figure 7b) revealed the complementary functional polarization between symbiotic and non-symbiotic performance groups. FS1 and FS7, the weakest symbiotic performers, showed the highest positive z-scores for ACC deaminase activity and siderophore production. DX1 and DX5 showed moderately positive z-scores across ACC deaminase, siderophore, and IAA. SN1 and SN8 showed strongly positive z-scores for IAA production and soluble phosphorus, but negative z-scores for ACC deaminase activity and siderophore. SJ1 and SJ11 were generally intermediate across all PGPR indicators. This inverse relationship between symbiotic efficiency and stress-tolerance-related PGPR traits (ACC deaminase, siderophore) across ecological groups is consistent with an ecological trade-off between nitrogen-fixation investment and capacity for abiotic stress mitigation.

The composite z-score ranking, integrating all symbiotic and non-symbiotic indicators, produced a clear hierarchical ordering of the eight strains (Figure 7c). SN1 achieved the highest composite z-score, followed by SN8; Songnen Plain strains were well separated from the other strains in the positive z-score range. SJ1 and SJ11 ranked third and fourth, with positive but lower composite scores. DX5 ranked fifth and DX1 sixth, both with composite scores near zero. FS7 and FS1 ranked seventh and eighth, with negative composite z-scores, reflecting their limited symbiotic performance despite specialized PGPR traits. The rank order follows the pattern SN1 > SN8 > SJ1 > SJ11 > DX5 > DX1 > FS7 > FS1, with the top four strains (Songnen Plain and Sanjiang Plain) consistently scoring higher than the mountain-area and northwest-arid strains in the composite evaluation. SN1 and SN8 ranked highest overall, based on their composite scores and their relatively strong performance across symbiotic dimensions (nitrogenase activity, total nitrogen, nodule dry weight) and select non-symbiotic dimensions (IAA production for SN1; phosphate solubilization for both). Within the middle tier, DX5 showed stronger PGPR trait expression than DX1, consistent with its identification as a functionally balanced supplementary candidate. FS1, despite ranking lowest overall, showed the highest ACC deaminase activity in the collection, suggesting possible utility as a specialized strain for early-season abiotic stress conditions in cold-region soybean production.

## 3. Discussion

### 3.1. Habitat-Driven Phenotypic Differentiation Across a Cold-Region Ecological Gradient

The partial correspondence between phenotypic cluster structure and ecological origin observed in this study is consistent with the hypothesis that long-term habitat selection may contribute to phenotypic differentiation among Heilongjiang soybean rhizobia. Previous studies have reported that rhizobial metabolic and stress-tolerance phenotypes can be shaped by soil nutrient status and habitat stability; however, most such work has focused on single-region surveys or simple geographic comparisons, without systematic analysis across a continuous ecological gradient. The present study extends this framework to a gradient spanning wetland, fertile plain, mountainous, and arid habitats within a single high-latitude region.

The gradient in phenotypic differentiation from compact, conservative clustering in the Sanjiang Plain to the most divergent clustering in the northwest arid region is interpretable within established ecological theory. The stable, uniform wetland habitat of the Sanjiang Plain imposes low directional selection pressure, allowing rhizobial populations to maintain high phenotypic homogeneity consistent with K-strategy stabilization. The nutrient-rich and microenvironmentally diverse Songnen Plain black soil reduces selection intensity while supporting greater within-population phenotypic variation. In contrast, the persistent, multi-dimensional stress conditions in mountain and arid regions may be associated with broader r-strategy phenotypes, with strains expanding their substrate utilization ranges and reinforcing stress resistance as survival strategies. This pattern may differ from the more stochastic variation reported in some temperate rhizobial systems, though direct comparisons are limited by methodological differences among studies.

The positive covariation between metabolic breadth and stress-tolerance capacity across strains suggests that Heilongjiang rhizobia may rely on integrated multi-trait adaptation rather than on specialization of individual traits. In high-latitude environments with short growing seasons and slow nutrient turnover, maintaining rhizosphere colonization and symbiotic competitiveness may require the coordinated optimization of metabolic flexibility and environmental resistance. This covariation pattern is consistent with the view that strains with narrow metabolic profiles tend to show limited stress tolerance, and vice versa, with potential implications for inoculant strain selection in cold-region production systems.

### 3.2. Nodule Quality as the Primary Determinant of Symbiotic Nitrogen Fixation Efficiency

The present data suggest that nodule number is a poor predictor of symbiotic nitrogen fixation efficiency, whereas nodule biomass indicators were more consistently associated with nitrogenase activity and plant nitrogen accumulation. This result challenges the common practice of using nodule number as a primary screening criterion for rhizobial inoculant strains [17,18,19]. In short-season, cold-region soybean production, where growing degree-days are limited, the metabolic cost of forming and maintaining many nodules may reduce per-nodule nitrogen-fixation efficiency by diverting photosynthate from nodule development [20]. A smaller number of well-developed, highly active nodules may therefore be more compatible with the carbon economy of cold-region soybean than many smaller, less active ones.

Strains from nutrient-rich, thermally moderate environments may invest more metabolic resources in nodule development and nitrogen fixation, while strains from harsher environments may prioritize stress resistance at some cost to symbiotic efficiency, though this interpretation remains correlational in the absence of direct metabolic evidence [21]. This framework has been described in tropical and subtropical legume-rhizobium systems but has received less systematic attention in high-latitude cold-region contexts. The positive associations among nitrogenase activity, plant biomass, and total nitrogen accumulation are consistent with these traits forming a functionally integrated symbiotic module. Nodule dry weight, nitrogenase activity, and total plant nitrogen content may therefore represent more informative and less redundant indicators for symbiotic evaluation of Heilongjiang soybean rhizobia, with potential applicability to screening programs targeting inoculant development for northeastern China.

### 3.3. Functional Specialization in Non-Symbiotic Plant Growth-Promoting Traits

The co-stratification of IAA synthesis and siderophore production across strains is broadly consistent with reports from temperate soybean rhizobial systems, where these traits have been identified as coordinated characters [22,23,24]. However, the marked stratification observed here, with FS1 and FS7 showing elevated expression of both traits while Songnen and Sanjiang strains showed only baseline levels, differs from the more uniform distribution typically reported in warm-region studies. This pattern may reflect the influence of seasonal iron availability in black soil environments, where low temperatures reduce iron bioavailability, potentially relaxing selection pressure for autonomous iron acquisition in strains more reliant on symbiotic nutrient-supply pathways [25,26].

The notably high ACC deaminase activity of FS1 may confer specific ecological advantages under cold-region spring sowing conditions, where seedlings frequently experience simultaneous cold and drought stress. ACC deaminase reduces plant ethylene levels under stress by cleaving the ethylene precursor ACC, thereby alleviating ethylene-induced growth inhibition [27,28].

The functional profile of DX5, broadly balanced across all four PGPR indicators, represents a phenotype less commonly reported among Heilongjiang soil rhizobia. Most previous studies have classified rhizobia as either functionally generalist or specialized, with generalist profiles more frequently reported in warm acidic soils [6,29,30]. The identification of a broadly capable PGPR strain from a cold, mountainous habitat is consistent with the hypothesis that ecological heterogeneity and multifactor nutrient limitation in that environment may have selected for functional breadth over specialization; if so, DX5 may be particularly useful in soils subject to multiple simultaneous growth-limiting factors.

### 3.4. Validity and Utility of the Two-Dimensional Evaluation Framework

The two-dimensional evaluation framework addresses two key limitations of conventional rhizobial screening: multicollinearity among symbiotic trait indicators and the lack of a standardized quantitative method for jointly evaluating symbiotic and non-symbiotic functional data. By organizing symbiotic indicators according to the biological hierarchy of nodule organogenesis–nitrogen fixation–plant nitrogen yield and supplementing them with four functionally independent PGPR indicators, the framework reduces redundancy and captures the principal mechanisms by which rhizobia promote plant performance under cold-region conditions.

The PCA-derived weight distribution is broadly interpretable from an ecological perspective. Six of the seven indicators (total plant nitrogen, siderophore production, nitrogenase activity, ACC deaminase activity, IAA synthesis capacity, and nodule dry weight; weights 0.146–0.181, Table S1) contributed comparably to PC1, indicating that symbiotic nitrogen-fixation traits and non-symbiotic stress/growth-promoting traits are of broadly similar importance in distinguishing strains along this axis, rather than falling into a strict primary-versus-intermediate hierarchy. Only phosphate solubilization capacity contributed appreciably less (weight 0.013), suggesting it may be more environment-specific than a universally critical function, given moderate phosphorus levels in black soils. Six of the seven indicators (total plant nitrogen, siderophore production, nitrogenase activity, ACC deaminase activity, IAA synthesis capacity, and nodule dry weight; weights 0.146–0.181, Table S1) contributed comparably to PC1, indicating that symbiotic nitrogen-fixation traits and non-symbiotic stress/growth-promoting traits are of broadly similar importance in distinguishing strains along this axis, rather than falling into a strict primary-versus-intermediate hierarchy. Only phosphate solubilization capacity contributed appreciably less (weight 0.013), suggesting it may be more environment-specific than a universally critical function, given moderate phosphorus levels in black soil performance benefits. The distinct functional profiles of the top-ranked strains SN1, showing high nitrogenase activity, and SN8, showing high nodule biomass, suggest that the framework can differentiate strains with complementary, potentially compatible functional traits. These strains may be promising candidates for further evaluation in multi-location field trials, which will be required before formulation as single or combined inoculants targeting soybean cultivation in the cold environments of northeastern China.

### 3.5. Limitations

Several limitations should be considered when interpreting the findings of this study. First, the strains were designated as rhizobia based on nodulation phenotype and morphological characteristics rather than molecular identification. Although all isolates formed effective nodules on soybean, molecular characterization (e.g., *16S rRNA* gene sequencing or multilocus sequence analysis) would provide more robust taxonomic confirmation. Second, the symbiotic nitrogen fixation and plant growth-promoting (PGPR) traits were evaluated under controlled pot conditions using a single soybean cultivar during one growing cycle. Consequently, the observed performance may not fully reflect responses under diverse environmental conditions, and multi-location, multi-season field trials will be required before these strains can be recommended as commercial inoculants. Third, the eight representative strains selected for detailed functional characterization were chosen according to phenotypic clustering and dendrogram position rather than by random sampling. Although this strategy was designed to maximize phenotypic diversity, it may not completely capture the full functional variation present in the 66-strain collection and could introduce selection bias. Fourth, stress-tolerance assays were conducted using in vitro plate-based methods, which do not fully reproduce the complex environmental conditions experienced by rhizobia in the rhizosphere or within host plants. Therefore, differences in stress tolerance may partially reflect variation in bacterial growth characteristics rather than physiological adaptation alone. Fifth, the apparent trade-off between stress tolerance and symbiotic performance is based on correlative evidence obtained from a limited number of representative strains and should not be interpreted as demonstrating a causal resource-allocation mechanism. Finally, the PCA-derived indicator weights and the composite evaluation framework were developed using the specific strain collection and ecological conditions examined in this study. Their applicability to other rhizobial populations, host genotypes, or agroecological regions should therefore be validated and, where necessary, recalibrated before broader application.

## 4. Materials and Methods

### 4.1. Rhizobial Strains and Soybean Cultivar

Sixty-six indigenous soybean rhizobial strains were isolated from fresh nodule tissue of major soybean cultivars grown across the four ecological regions of Heilongjiang Province, China. Following isolation, purification, colony morphology assessment, Gram staining, and host plant reinoculation, all strains were confirmed to form effective symbiotic nodules with soybean. Strains are maintained on YMA (Yeast Mannitol Agar) slant medium at 4 °C in the Key Laboratory of Soybean Cultivation at the Soybean Research Institute, Heilongjiang Academy of Agricultural Sciences. Strains were classified into four ecological groups based on collection site: Songnen Plain (SN1–SN24, n = 24), Sanjiang Plain (SJ1–SJ22, n = 22), northwest arid sandy region (FS1–FS10, n = 10), and Daxing’anling–Xiaoxing’anling mountainous area (DX1–DX10, n = 10). The soybean cultivar Heinong 507, a widely adapted elite cultivar that performs consistently across all four ecological regions of Heilongjiang Province, served as the uniform host for all pot experiments. Uniform, fully filled seeds were surface sterilized prior to sowing (75% ethanol, 30 s; 0.1% HgCl_2_, 5 min; thorough rinsing with sterile water).

### 4.2. Assessment of Carbon and Nitrogen Source Utilization

Carbon and nitrogen source utilization was assessed on YMA basal salt medium with the default carbon or nitrogen source omitted. For carbon source utilization, malic acid, inositol, creatine, sucrose, glucose, D-fructose, and lactose were each substituted for mannitol as the sole carbon source at 10 g L^−1^. For nitrogen source utilization, L-tryptophan, glycine, arginine, L-histidine, and phenylalanine were each substituted for tryptone as the sole nitrogen source at 10 g L^−1^. Log-phase cultures were adjusted to OD_600_ = 0.80 ± 0.02, and 5 µL aliquots were spot-inoculated onto selective solid media. Plates were incubated at 28 °C for 3 days and then inverted. Uninoculated medium served as the negative control. Three biological replicates were performed per treatment. Visible colony growth was scored as positive; absence of visible growth was scored as negative.

### 4.3. Stress Tolerance Assays

Salt tolerance was evaluated on YMA supplemented with NaCl at 0.5%, 1.0%, 1.5%, 2.0%, and 2.5% (*w*/*v*). Acid-alkali tolerance was assessed on YMA adjusted to pH 4.0, 5.0, 6.0, 7.0, 8.0, 9.0, and 10.0 using sterile NaOH. Temperature tolerance was evaluated by incubating inoculated plates at 4, 10, 28, 37, and 45 °C; 28 °C served as reference control. Drought tolerance was assessed using YMA supplemented with PEG-6000 at 10%, 15%, 20%, 25%, and 30% (*w*/*v*). Standardized inocula (OD_600_ = 0.80 ± 0.02; 2 µL) were spot inoculated onto plates and incubated under the respective conditions for 7 days. Visible colony formation was scored as positive; absence of visible growth was scored as negative. Three biological replicates were included per treatment.

### 4.4. Physiological and Biochemical Characterization

Eight standard physiological and biochemical tests were performed according to established protocols: starch hydrolysis, gelatin liquefaction, hydrogen sulfide production, indole production, the BTB (bromothymol blue) acid-alkali reaction, citrate utilization, the Voges-Proskauer (VP) test, and catalase activity. Standardized inocula (OD_600_ = 0.80 ± 0.02) were used for all tests except the catalase test, which used freshly picked single colonies. Positive and negative criteria were applied in accordance with established microbiological identification protocols (Bergey’s Manual of Systematic Bacteriology). Three biological replicates were performed per test.

### 4.5. Cluster Analysis

All phenotypic data were binary-coded (positive = 1, negative = 0) to build a phenotypic trait matrix. Hierarchical clustering was performed using Jaccard distance and the unweighted pair-group method with arithmetic mean (UPGMA). A phenotypic dendrogram was generated, and strains were assigned to functional groups at a cluster distance threshold of 10. A heatmap was constructed to visualize phenotypic similarity patterns and their correspondence with ecological origin.

### 4.6. Pot Experiment: Symbiotic Nitrogen Fixation

Symbiotic nitrogen-fixation capacity was evaluated using a sand-culture pot system. The quartz sand substrate was autoclaved at 121 °C for 30 min. Soybean seeds were sown in standardized pots after surface sterilization. After the first true leaf emerged, inocula adjusted to an OD_600_ of approximately 1.0 were applied by ring-trench inoculation (20 mL per plant, 2–3 cm from the main root). An uninoculated blank control was included. Three biological replicates were established per strain. Plants were grown under a 14 h/10 h (light/dark) photoperiod at approximately 300 µmol m^−2^ s^−1^ photosynthetically active radiation, 26 °C/22 °C day/night temperature, and 60–70% relative humidity. A nitrogen-free nutrient solution was supplied every 3 days. Plants were harvested 45 days after inoculation. At harvest, the following parameters were measured: nodule number per plant, nodule fresh weight, and nodule dry weight (oven-dried at 65 °C to constant weight after killing at 105 °C for 30 min); plant height; shoot and root fresh and dry weights; nitrogenase activity by the acetylene reduction assay (0.2 g fresh nodules per sample in sealed vials with 1 mL acetylene at 28 °C for 2 h in darkness; ethylene quantified by gas chromatography and expressed as nmol C_2_H_4_ g^−1^ h^−1^); and total plant nitrogen content by the Kjeldahl method (expressed as mass fraction, %).

### 4.7. Plant Growth-Promoting (PGPR) Trait Assays

Inorganic phosphate solubilization capacity was assessed by inoculating strains (2% *v*/*v*) into 50 mL of BAP liquid medium and incubating at 30 °C and 180 rpm for 7 days. Supernatant was collected after centrifugation (12,000 rpm, 10 min) and soluble phosphorus quantified at 660 nm by the molybdenum-antimony colorimetric method using potassium dihydrogen phosphate as the standard (mg L^−1^). Indole Acetic Acid (IAA) synthesis capacity was assessed by inoculating strains (2% *v*/*v*) into a liquid medium supplemented with L-tryptophan (0.5 mg L^−1^) and incubating at 30 °C and 180 rpm for 5 days. After centrifugation (12,000 rpm, 10 min), 2 mL of supernatant was reacted with 4 mL Salkowski reagent in the dark for 30 min, and the absorbance was measured at 530 nm. IAA concentration was calculated from a standard curve and expressed as µg mL^−1^.

1-aminocyclopropane-1-carboxylate (ACC) deaminase activity was measured using a two-step protocol. Strains were pre-cultured in DF liquid medium (30 °C, 180 rpm, 24 h), then transferred to ADF medium (1% *v*/*v*) for 48 h. Cell pellets (0.5 g wet weight) were disrupted by sonication on ice (200 W, 3 s on/5 s off, 30 cycles) to obtain crude enzyme extract. α-Ketobutyrate production was measured at 540 nm, and activity was expressed as nmol α-ketobutyrate g^−1^ (wet cells) h^−1^. Siderophore production was measured by a modified chrome azurol S (CAS) liquid colorimetric assay [31]. Standardized inocula (OD_600_ = 0.8) were cultured in iron-free MSA liquid medium (28 °C, 180 rpm, 72 h). After centrifugation (10,000 rpm, 15 min, 4 °C), 2 mL of supernatant was mixed with 2 mL of CAS solution and 0.5 mL of shuttle solution and left at room temperature in the dark for 60 min. Absorbance was measured at 630 nm. Siderophore relative production rate (%) = (A_0_ − As)/A_0_ × 100%, where A_0_ and As are blank and sample absorbances, respectively. All PGPR assays were performed in eight biological replicates.

### 4.8. Comprehensive Evaluation Framework

A two-dimensional evaluation framework integrating symbiotic and non-symbiotic functional traits was constructed. To minimize multicollinearity, three symbiotic core indicators were selected following the biological hierarchy of nodule organogenesis–nitrogen fixation expression–plant nitrogen yield: nodule dry weight, nitrogenase activity, and total plant nitrogen content. Four non-symbiotic growth-promoting indicators were included: IAA synthesis capacity, siderophore production rate relative to total production, ACC deaminase activity, and inorganic phosphate solubilization capacity. Siderophore production rate and ACC deaminase activity were subjected to directional correction (reverse normalization) because their raw measurement scales were inverted relative to the desired scoring direction: higher raw values for these traits were associated with greater abiotic stress exposure rather than with symbiotic performance. This step ensured directional consistency across all indicators in the composite index and does not imply these traits are biologically detrimental. Principal component analysis was applied to calculate indicator weights and derive composite strain scores for ranking.

### 4.9. Statistical Analysis

All data were analyzed using R (version 4.3.1; R Core Team, 2023) and SPSS Statistics 26.0 (IBM Corp., Armonk, NY, USA). For pot experiments, each pot containing a single inoculated plant constituted one biological replicate; three independent pots per strain were used (n = 3). Technical measurements (e.g., nitrogenase activity) were averaged within each biological replicate before entry into statistical analyses. For PGPR trait assays, eight biological replicates were performed per strain. Differences among strain means were assessed by one-way ANOVA followed by Duncan’s multiple range test at *p* < 0.05. Pearson correlation coefficients were calculated for pairwise trait associations. Hierarchical cluster analysis used Ward’s linkage with Euclidean distance (R hclust function). PCA was performed on z-score-standardized data matrices using the prcomp function in R.

## 5. Conclusions

This study characterized the phenotypic and functional diversity of 66 indigenous soybean rhizobia from four ecological regions of Heilongjiang Province. Hierarchical clustering revealed a differentiation gradient, ranging from relatively uniform profiles in the Sanjiang Plain to markedly divergent profiles in the northwest arid region, consistent with increasing habitat stress intensity. Positive covariation between metabolic breadth and stress-tolerance capacity suggests that multi-trait adaptation may be a general feature of Heilongjiang rhizobia populations. Pot experiment results indicated that nodule number was a poor predictor of nitrogen fixation efficiency; nodule dry weight, nitrogenase activity, and total plant nitrogen content appeared more informative and are proposed as primary screening criteria. Symbiotic performance broadly followed the regional ecological gradient, consistent with a trade-off between stress resistance and symbiotic investment. A two-dimensional, seven-parameter evaluation framework provided a quantitative basis for ranking strains, with symbiotic nitrogen fixation representing the primary performance dimension, stress-tolerance-related PGPR traits contributing secondary functional stability, and nutrient-mobilizing traits offering context-specific supplementary benefits. SN1 and SN8 emerged as high-priority candidates for inoculant development based on their symbiotic performance rankings, though multi-environment field validation will be required before commercial application; FS1 showed potential for specialized application under early-season abiotic stress conditions, and DX5 displayed a comparatively broad PGPR functional profile. Together, these findings may help clarify ecological drivers of rhizobial adaptation in cold-region systems and offer a practical screening framework to support region-specific inoculant development for sustainable soybean production in northeastern China.

## Figures and Tables

**Table 1:** Carbon and nitrogen source utilization rates (%) across four ecological regions of Heilongjiang Province.

Type	Substrate	Songnen Plain (*n* = 24)	Sanjiang Plain (*n* = 22)	NW Arid (*n* = 10)	Mountain (*n* = 10)	All Strains (*n* = 66)
Carbon	Malic acid	100.00	95.45	100.00	100.00	98.48
	Glucose	100.00	90.91	100.00	90.00	95.45
	D-Fructose	100.00	95.45	100.00	100.00	96.97
	Sucrose	95.83	90.91	100.00	90.00	90.91
	Inositol	91.67	86.36	90.00	80.00	87.88
	Creatine	95.83	90.91	100.00	90.00	89.39
	Lactose	91.67	72.73	90.00	80.00	86.36
	L-Tryptophan	95.83	90.91	100.00	80.00	90.91
Nitrogen	Glycine	91.67	86.36	90.00	80.00	87.88
	Arginine	100.00	95.45	100.00	100.00	98.48
	L-Histidine	79.17	81.82	90.00	90.00	84.85
	Phenylalanine	95.83	95.45	90.00	100.00	96.97

NW Arid = Northwest Arid Sandy Region; Mountain = Daxing’anling–Xiaoxing’anling Eastern Hilly Area. Values are the proportion (%) of strains in each region that show positive growth.

**Table 2:** Proportion of strains (%) positive for stress tolerance indicators across four ecological regions.

Stress Indicator	Songnen Plain	Sanjiang Plain	NW Arid	Mountain
NaCl 0.5%	100	100	100	100
NaCl 1.0%	100	100	100	100
NaCl 1.5%	95.7	91.3	100	96.3
NaCl 2.0%	73.9	65.2	92.9	77.8
NaCl 2.5%	13.0	4.3	42.9	14.8
pH 4.0	21.7	13.0	57.1	25.9
pH 5.0	91.3	87.0	100	92.6
pH 6.0–8.0	100	100	100	100
pH 9.0	95.7	91.3	100	96.3
pH 10.0	34.8	26.1	71.4	37.0
4 °C	0	0	0	0
10 °C	13.0	8.7	14.3	29.6
28 °C (control)	100	100	100	100
37 °C	82.6	73.9	92.9	85.2
45 °C	4.3	0	21.4	7.4
PEG-6000 10%	100	100	100	100
PEG-6000 15%	95.7	91.3	100	96.3
PEG-6000 20%	47.8	34.8	78.6	51.9
PEG-6000 25%	8.7	4.3	35.7	11.1
PEG-6000 30%	0	0	7.1	0

NW Arid = Northwest Arid Sandy Region; Mountain = Daxing’anling–Xiaoxing’anling Eastern Hilly Area.

**Table 3:** Proportion of strains (%) positive for physiological and biochemical tests across four ecological regions.

Test	Songnen Plain	Sanjiang Plain	NW Arid	Mountain
**Starch hydrolysis**	52.2	43.5	71.4	55.6
**Gelatin liquefaction**	47.8	39.1	64.3	51.9
**H_2_S production**	8.7	4.3	21.4	11.1
**Indole production**	13.0	8.7	28.6	14.8
**BTB alkaline reaction**	91.3	87.0	100	92.6
**Citrate utilization**	82.6	73.9	92.9	85.2
**VP reaction**	17.4	13.0	35.7	18.5
**Catalase**	100	100	100	100

NW Arid = Northwest Arid Sandy Region; Mountain = Daxing’anling–Xiaoxing’anling Eastern Hilly Area.

## Data Availability

The data supporting the findings of this study are available from the corresponding author upon reasonable request.

## Supplementary Materials

The following supporting information can be downloaded at: https://www.mdpi.com/article/10.3390/plants15152251/s1, Table S1: Principal component 1 (PC1) loadings and derived weights for the seven indicators used in the two-dimensional evaluation framework; Table S2: Composite PC1-based scores and rankings for the eight strains selected for detailed symbiotic and PGPR trait profiling; Table S3: Variance explained by all seven principal components.
