# Supplementary material for: Multi-Trait Screening Identifies Cold-Adapted Soybean Rhizobia from Northeastern China as Candidate Inoculant Strains
Source: Plants (Basel). 2026 Jul 23;15(15):2251. doi: 10.3390/plants15152251 (PMC13468475; doi:10.3390/plants15152251)
Supplement: Supplementary file 1 [file plants-15-02251-s001.zip › plants-4428027-supplementary.pdf]

# Supplementary Tables

**Table S1. Principal component 1 (PC1) loadings and derived weights for the seven indicators used in the two-dimensional evaluation framework.**

| Indicator | PC1 loading | Weight |
|---|---|---|
| Nodule dry weight | 0.382 | 0.146 |
| Nitrogenase activity | 0.413 | 0.171 |
| Total plant nitrogen content | 0.425 | 0.181 |
| IAA synthesis capacity | 0.384 | 0.148 |
| Siderophore production (reverse-normalized) | −0.418 | 0.175 |
| ACC deaminase activity (reverse-normalized) | −0.408 | 0.167 |
| Phosphate solubilization capacity | 0.115 | 0.013 |

*Weights were derived from squared PC1 loadings, normalized to sum to 1. Negative loadings for siderophore production and ACC deaminase activity reflect their natural covariation with the more ecologically stressed strains in this dataset, consistent with the reverse-normalization described in Section 4.8; these values do not indicate that either trait is biologically detrimental to overall strain performance.*

**Table S2. Composite PC1-based scores and rankings for the eight strains selected for detailed symbiotic and PGPR trait profiling.**

| Strain | Composite score (0–100) | PC1 score | Rank | Ecological region |
|---|---|---|---|---|
| SN1 | 100.00 | 3.042 | 1 | Songnen Plain |
| SN8 | 86.08 | 2.187 | 2 | Songnen Plain |
| SJ1 | 71.51 | 1.292 | 3 | Sanjiang Plain |
| SJ11 | 67.57 | 1.050 | 4 | Sanjiang Plain |
| DX5 | 42.93 | −0.463 | 5 | Khingan Mountains |
| DX1 | 35.02 | −0.949 | 6 | Khingan Mountains |
| FS1 | 0.67 | −3.059 | 7 | Northwest Area |
| FS7 | 0.00 | −3.100 | 8 | Northwest Area |

*Composite scores were rescaled to a 0–100 range for interpretability, with 100 representing the highest-ranked strain (SN1). PC1 score is the unscaled principal component score.*

**Table S3. Variance explained by all seven principal components.**

| Component | Variance explained (%) | Cumulative (%) |
|---|---|---|
| PC1 | 75.28 | 75.28 |
| PC2 | 15.97 | 91.26 |
| PC3 | 5.70 | 96.95 |
| PC4 | 2.14 | 99.09 |
| PC5 | 0.70 | 99.79 |
| PC6 | 0.20 | 99.99 |
| PC7 | 0.01 | 100.00 |

*PC1 alone accounts for 75.3% of total variance among the seven indicators, well above conventional thresholds (e.g., Kaiser criterion, scree-plot inflection) for justifying its use as a single composite ranking axis. PC2 is dominated by phosphate solubilization capacity (loading 0.908) and explains an additional 16.0% of variance; components beyond PC2 individually explain less than 6% each and were not considered further.*
